# Human insulin analogues modified at the B26 site reveal a hormone conformation that is undetected in the receptor complex

**DOI:** 10.1107/S1399004714017775

**Published:** 2014-09-27

**Authors:** Lenka Žáková, Emília Kletvíková, Martin Lepšík, Michaela Collinsová, Christopher J. Watson, Johan P. Turkenburg, Jiří Jiráček, Andrzej M. Brzozowski

**Affiliations:** a Institute of Organic Chemistry and Biochemistry, Academy of Sciences of the Czech Republic, v.v.i., Flemingovo nám. 2, 166 10 Prague 6, Czech Republic; b York Structural Biology Laboratory, Department of Chemistry, The University of York, Heslington, York YO10 5DD, England

**Keywords:** insulin, insulin receptor, complex, active conformation, molecular dynamics, isothermal titration microcalorimetry

## Abstract

The structural characterization of the insulin–insulin receptor (IR) interaction still lacks the conformation of the crucial B21–B30 insulin region, which must be different from that in its storage forms to ensure effective receptor binding. Here, it is shown that insulin analogues modified by natural amino acids at the TyrB26 site can represent an active form of this hormone. In particular, [AsnB26]-insulin and [GlyB26]-insulin attain a B26-turn-like conformation that differs from that in all known structures of the native hormone. It also matches the receptor interface, avoiding substantial steric clashes. This indicates that insulin may attain a B26-turn-like conformation upon IR binding. Moreover, there is an unexpected, but significant, binding specificity of the AsnB26 mutant for predominantly the metabolic B isoform of the receptor. As it is correlated with the B26 bend of the B-chain of the hormone, the structures of AsnB26 analogues may provide the first structural insight into the structural origins of differential insulin signalling through insulin receptor A and B isoforms.

## Abstract 2

[AsnB26]- and [GlyB26]-insulin mutants attain a B26-turn like fold without assistance of chemical modifications. Their structures match the insulin receptor interface and expand the spectrum of insulin conformations.

## 1. Introduction   

Understanding of the insulin–insulin receptor (IR) interaction is one of the key issues in human physiology and its clinical manipulation. Insulin is a 51-amino-acid protein hormone with two disulfide-linked chains (A1–A21 and B1–B30) that is responsible for the maintenance of metabolic homeostasis, with an impact on cell proliferation and regulation of ageing (Taniguchi *et al.*, 2006 ▶; Cohen, 2006 ▶). It exerts its functions through binding as a monomer to the tyrosine kinase-type IR (Lemmon & Schlessinger, 2010 ▶), which is an (αβ)_2_ heterotetramer in which each individual αβ extracellular chain comprises L1, CR, L2, FnIII-1, FnIII-2 and FnIII-3 domains (McKern *et al.*, 2006 ▶). The first three-dimensional structure of insulin determined by D. Hodgkin in 1969 (Adams *et al.*, 1969 ▶) was followed by many storage-form (hexamers and dimers) structures of this hormone and its analogues (De Meyts & Whittaker, 2002 ▶). Two surfaces on the insulin molecule that are responsible for effective IR binding have also been identified (with the main site 1 comprising GlyA1–ValA3, GlnA5, TyrA19, LeuB11, ValB12, GlnB15, PheB24 and PheB25 and site 2 comprising SerA12, LeuA13, GluA17, HisB10, GluB13 and LeuB17; De Meyts & Whittaker, 2002 ▶; Menting *et al.*, 2013 ▶). It also became apparent that insulin must undergo a substantial structural change upon IR binding to fully expose site 1, with the B-chain C-terminus (B25–B30) leaving the insulin core to uncover key IR binders obstructed by this segment in its storage forms (Dodson *et al.*, 1983 ▶; Hua *et al.*, 1991 ▶). It has also been shown that residues B26–B30 are not important for IR binding. Deletion of the B26–B30 segment (provided that the C-terminal PheB25 is carboxy-amidated) does not change the IR affinity of the hormone (Fischer *et al.*, 1985 ▶).

Extensive mutagenesis and cross-linking studies of both insulin and IR led to the discovery and mapping of two sites on the IR that are responsible for engaging with the hormone. The so-called main binding site 1, with a *K*_d_ of ∼6.4 n*M*, involves the L1 domain of one α subunit and a 19-amino-acid αCT segment from the C-terminal end of the other α subunit. Furthermore, the junction of the FnIII-1 and FnIII-2 domains of this other α subunit contribute to the insulin-binding low-affinity site 2, which has a *K*_d_ of ∼400 n*M* (Kiselyov *et al.*, 2009 ▶). The hormone–receptor interaction events are complicated further by the tissue-specific expression of two isoforms of the IR, IR-A and IR-B, which differ by an extra 12 amino acids (coded by an alternatively spliced exon 11) that are present in the above-mentioned C-terminal αCT segment of the IR-B isoform. Moreover, IR-A is also a relatively good binder (*K*_d_ of ∼4 n*M*; Morcavallo *et al.*, 2012 ▶) of insulin-like growth factor II (IGF-II), which is a single-polypeptide mitogenic hormone with an insulin-like structure. IGF-II, and its closely related hormone IGF-I, has its own IGF-1R receptor, which is highly homologous to IR. Consequently, the heterodimerization of all of these receptors, resulting in IR/IGF-1R hybrids, further expands the already complex network of insulin/IGF signalling pathways (Sciacca *et al.*, 2012 ▶).

Formidable problems with the production of IR constructs for structural studies, and the complexity of IR–insulin interaction, interfered with the progress of deciphering the molecular nature of this assembly. Therefore, only the structures of the apo forms of some IR and IGF-1R ecto­domain constructs were available prior to 2013 (McKern *et al.*, 2006 ▶; Garrett *et al.*, 1998 ▶).

**Figure 1:**
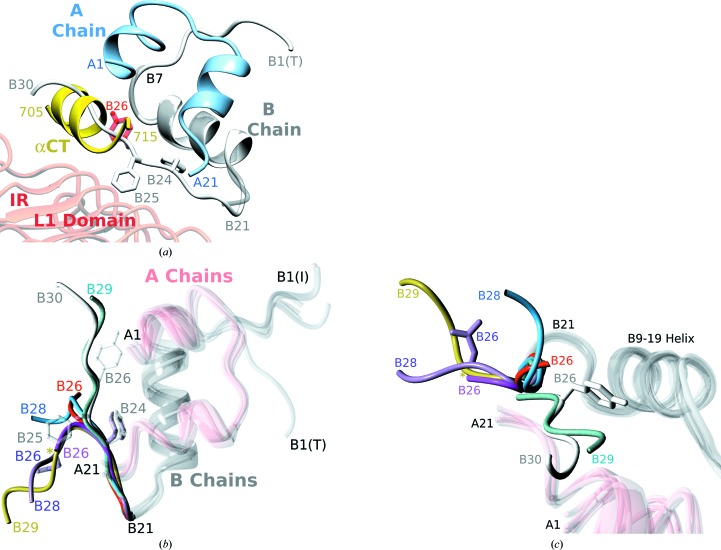
(*a*) Conformational incompatibility of the B25–B30 chain of native insulin (in white; PDB entry 1mso) with human IR. Native (hexamer-derived; PDB entry 1mso) insulin is superimposed on the invariant B9–B19 helix (in grey) in the insulin–IR complex; only the A-chain of IR-complexed insulin is shown for clarity. The TyrB26 site mutated in this work is highlighted in red. (*b*) Appearance of the B26-turn-like B-chain conformation in native insulin (PDB entry 1mso; white) and the insulin analogues [PheB26]-insulin (sea blue), [GlyB26]-insulin (gold), [AsnB26]-insulin (lilac), [d-ProB26]-DTI-NH_2_ insulin (red; Jiracek *et al.*, 2010 ▶), [d-AlaB26]-DTI-NH_2_ insulin (magenta; Jiracek *et al.*, 2010 ▶) and [NMeAlaB26]-insulin (blue; Jiracek *et al.*, 2010 ▶). Only the B21–B30 parts of the B-chains are colour-coded; ‘invariant’ A-chains and B-chains are shown in pink and grey, respectively. B1(T) indicates the T-­state of native human insulin, while B1(I) denotes the so-called intermediate conformation of the B-chain N-termini. Only the B24–B26 side chains of native insulin are shown; PheB24 for [AsnB26]-insulin is shown as an example of the conservation of this side-chain conformation in all analogues shown here; a gold asterisk indicates the GlyB26 site in [GlyB26]-insulin. (*c*) The conformational spread of the B25–B30 insulin chain in wild-type insulin and the B26-turn- and B26-bend-containing analogues. Colour coding is as in (*b*). Only the B21–B30 parts of the B-chains are colour-coded; A-chains are shown in pink and B-chains in grey; a gold asterisk indicates the position of GlyB26 in [GlyB26]-insulin. The positions of the B26 side chains in wild-type insulin (TyrB26; white) and [AsnB26]-insulin (B26Asn; lilac) are also shown.

The long-awaited first structural insight into the insulin–IR complex provided a breakthrough in the understanding of the nature of this interaction (Menting *et al.*, 2013 ▶). It showed that insulin site 1 is engaged with its IR counterpart (IR site 1) predominantly *via* the IR αCT helical segment (the 704–719 helix), which is tethered across the β-sheet of the L1 domain (Fig. 1 ▶ *a*). The helical αCT peptide undergoes a shift and remodelling from the apo IR to the holo IR to mediate the connection of the IR L1 domain with the hormone (Whittaker *et al.*, 2012 ▶; Menting *et al.*, 2013 ▶). The αCT–insulin interface is fulfilled by only the A-chain site residues of the hormone, while direct L1–insulin A-chain contacts are limited. The central insulin B-chain helix forms multiple contacts with the L1 domain. However, the important B21–B30 part of the B-chain of insulin is not visible in the current complex structures (Menting *et al.*, 2013 ▶). Nevertheless, it was clear that the B21–B30 β-strand must change its conformation from its storage-like fold to expose site 1 and to avoid a clash with the αCT segment of the IR (Fig. 1 ▶ *a*). The nature of the B-chain N-terminus (B1–B8), which in storage forms is generally found in the so-called T-state (extended) or R-state (helical, expanding the B9–19 helix), also remains undetermined in these complexes. However, our recent findings indicate that neither classical R- or T-states fulfil the criteria for an active hormone conformation and that high flexibility of the B-chain N-terminus is crucial for full hormone activity (Kosinová *et al.*, 2013 ▶).

In the absence of a clear structural definition of the B21–B30 insulin chain on the IR, two alternative models have been proposed for conformational structural ‘activation’ of insulin in the IR interface. The first model, which was based on mutagenesis of insulin and IR and photo-cross-linking with IR, involves full detachment of the B21–B30 segment (Hua *et al.*, 2009 ▶), including the important PheB24 and PheB25 residues. The second model, which was based on nonstandard modifications of the B24–B26 sites, results from a clear correlation between high affinity of the insulin analogue and the occurrence of the so-called B26 turn, in which only the B26–B30 segment of the B-chain departs from the core of the hormone (Jiracek *et al.*, 2010 ▶; Žáková *et al.*, 2013 ▶), with the B24 site remaining practically invariant in this process. Therefore, the exact character of the conformational switch of the B21–B30 chain of insulin in IR-mediated signal transduction, one of the key features of this process, remains unclear.

Therefore, we undertook further mutagenesis-driven studies of insulin, focusing on the TyrB26 site that we assumed might be the critical structural pivot of insulin conformational activation on its receptor. Our previous work on the modification of the B26 site resulted in several high-affinity analogues (Jiracek *et al.*, 2010 ▶). They contained an *N*-methylated B25–B26 peptide bond or d-amino acids instead of the natural TyrB26. Their high affinities (∼200–400%) also required the deletion of the B27–B30 chain and the presence of the B26 carboxamide C-terminus. All of these ‘superactive’ analogues possess a structural signature (the B26 turn) in which the B26–B30 chain bends away from the insulin core. This exposes site 1 residues that become primed for effective engagement with the IR, while the B20–B25 segment of the B-chain remains practically unchanged in this transition.

Here, we probe whether this conformational change can be achieved without any chemical modification but solely by natural amino-acid substitutions in order to provide a clearer picture of the physiological relevance of the B26 turn in the context of the currently determined insulin–IR structure.

## 2. Materials and methods   

### 2.1. Synthesis of analogues   

Solid-phase synthesis of peptides, enzymatic semisynthesis of analogues and purification of analogues were performed as described in detail previously (Žáková *et al.*, 2008 ▶).

### 2.2. Receptor-binding studies   

The binding data for IM-9 cells and mouse embryonic fibroblasts reported here (see below) were analyzed and the dissociation constants (*K*_d_) were determined with *GraphPad Prism* 5 using a nonlinear regression method, a one-site fitting program and taking into account potential depletion of free ligand. However, binding data for the IM-9 cells (the IR-A isoform) were also analyzed using *Excel* software developed especially for the IM-9 cell system in the laboratory of Professor Pierre De Meyts (A. V. Groth & R. M. Shymko, Hagedorn Research Institute, Denmark; a kind gift from P. De Meyts), which also uses a one-site fitting approach and potential ligand depletion. Although both methods gave fully convergent results that were in perfect agreement, the data given for the IR-A isoform in Table 2 correspond to the Hagedorn program-derived *K*_d_ estimations, as this is currently the optimum approach for binding analysis of the IM-9 IR-expressing system.

#### 2.2.1. Human IM-9 lymphocytes (human IR-A isoform)   

Receptor-binding studies with the insulin receptor in membranes of human IM-9 lymphocytes (containing only the human IR-A isoform) were performed and *K*_d_ values were determined according to the procedure described recently by Morcavallo *et al.* (2012 ▶).

#### 2.2.2. Mouse embryonic fibroblasts (human IR-B isoform)   

Receptor-binding studies with the insulin receptor in membranes of mouse embryonic fibroblasts derived from IGF-1 receptor knockout mice (Sell *et al.*, 1994 ▶) expressing solely the human IR-B isoform (Miura *et al.*, 1995 ▶; Frasca *et al.*, 1999 ▶) were generally performed according to Frasca *et al.* (1999 ▶). The cells were a kind gift from Professor Antonino Belfiore (University of Magna Grecia, Catanzaro, Italy) and Professor Renato Baserga (Thomas Jefferson University, Philadelphia, Pennsylvania, USA). Briefly, the fibroblasts expressing solely the IR-B isoform were cultured in DMEM medium with 5 m*M* glucose supplemented with 10% fetal bovine serum, 2 m*M* l-glutamine, 100 U ml^−1^ penicillin, 100 µg ml^−1^ streptomycin, 3 µg ml^−1^ puromycin in humidified air with 5% CO_2_ at 37°C. 2 d before testing, the cells were seeded into 24-well plates (Schoeller; about 3000 cells per well). The cells were grown to approximately 80% confluence (about 35 000–40 000 cells per well). For the assay, the cells were washed twice with binding buffer (100 m*M* HEPES–NaOH pH 7.6, 100 m*M* NaCl, 5 m*M* KCl, 1.3 m*M* MgSO_4_, 1 m*M* EDTA, 10 m*M* glucose, 15 m*M* sodium acetate, 1% bovine serum albumin). The cells were then incubated and shaken for 16 h at 5°C with increasing concentrations of human insulin or analogue and 43 p*M* ^125^I-TyrA14 human insulin (2200 Ci mmol^−1^; PerkinElmer) in a total volume of 0.25 ml binding buffer. The cells were washed twice with ice-cold binding buffer and solubilized with 0.1 *M* NaOH. The solutions of solubilized cells were measured for cell-associated radioactivity using a Wizard 1470 Automatic Counter (PerkinElmer Life Sciences). The binding data were analyzed and the dissociation constant (*K*_d_) was determined with *GraphPad Prism* 5 using a nonlinear regression method, a one-site fitting program and taking into account potential depletion of free ligand. Each binding curve was determined at least in duplicate. The dissociation constant of human ^125^I-insulin was 0.3 n*M*.

#### 2.2.3. Rat adipocyte membranes (both rat IR-A and IR-B isoforms)   

Receptor-binding studies with the insulin receptor in plasma membranes containing both rat IR-A and IR-B isoforms prepared from epididymal adipose tissue of adult male Wistar rats were performed and analyzed according to Žáková *et al.* (2008 ▶). Binding data were analyzed using *GraphPad Prism* 5. Half-maximal inhibition values of binding of [^125^I]-insulin to the receptor (IC_50_) were obtained using a nonlinear regression method and a one-site fitting program.

### 2.3. Isothermal microcalorimetry (ITC) measurements   

Investigations of the dimerization abilities of selected insulin analogues were performed as described in detail previously (Antolikova *et al.*, 2011 ▶).

### 2.4. Molecular modelling   

#### 2.4.1. Structure preparation   

The crystal structure of an insulin dimer from its hexameric oligomer (PDB entry 1mso; Smith *et al.*, 2003 ▶) was used as a starting model. Both of the crystallo­graphically independent *A*/*B* and *C*/*D* monomers were considered here. In cases of alternative conformations of the side chains (named *A* or *B*, not to be confused with the insulin *A*/*B* monomers), the *A* conformers were selected. All waters were discarded except for the molecule that bridges the TyrB26 OH group with the CO or alternatively the NH of GlyB8 (W609 and W623 for the *A*/*B* and *C*/*D* monomers, respectively), which is referred to here as Wat1. H atoms were added using *Reduce* (Word *et al.*, 1999 ▶) and the *LEaP* module of the *AMBER* 10 suite (Case, 2008 ▶) and were relaxed using a short high-temperature molecular-dynamics (MD) simulation (2 ps at 2400 K followed by cooling to 0 K during 8 ps). Histidine residues were located on the insulin periphery and were modelled as neutral and monoprotonated on N^∊^, except for HisB5 in the *C*/*D* monomer, which was protonated on N^δ^ owing to its hydrogen bond to Cys7 O. All Asp, Glu, Arg and Lys residues, as well as the N- and C-termini, were charged. The side chain of PheB1 of the *C*/*D* monomer lacked experimental electron density and was thus modelled as Ala. In both the *A*/*B* and *C*/*D* monomers there were three disulfide bridges: Cys*A*6–Cys*A*11, Cys*A*7–Cys*B*7 and Cys*A*20–Cys*B*19. Amino-acid parameters were taken from the ff99SB AMBER force field (Hornak *et al.*, 2006 ▶). The mutations at residue B26 were introduced by truncating the wild-type TyrB26 side chain and adding the missing atoms in *LEaP*. Subsequently, the B26 residue and Wat1 were subjected to a 10 ps MD simulation, warming from 10 to 300 K in 2 ps, followed by cooling to 0 K.

#### 2.4.2. Interaction energy calculations   

The prepared structures of TyrB26, PheB26 and AsnB26 insulin monomer variants were optimized using the corrected semiempirical quantum-chemical method PM6-D3H4 (Řezáč *et al.*, 2009 ▶; Řezáč & Hobza, 2012 ▶) in implicit COSMO solvent (Klamt & Schuurmann, 1993 ▶) of ∊_r_ = 78.4 to mimic the water environment. The *MOPAC*2009 program with the *MOZYME* linear-scaling algorithm was employed (Stewart, 2009 ▶, 2013 ▶).

The convergence criteria for optimization were a maximum energy change Δ*E* = 0.006 kcal mol^−1^, a maximum gradient of 1.2 kcal mol^−1^ Å^−1^ and a root-mean-square of the gradient of 0.6 kcal mol^−1^ Å^−1^. For the interaction energy calculations, the main chain of residue B26 and the whole residue B27 were removed and the cut bonds were capped with H atoms. The structural water (Wat1) was included as part of the protein only for the native insulin, while it was discarded for the other mutated variants. The interaction energy was calculated as the difference between the energy of the complex and the sum of the energies of the constituents at the PM6-D3H4 level in COSMO implicit solvent.

### 2.5. X-ray crystallography   

The crystallizations of all of the insulin analogues reported here were performed with in-house insulin crystallization screens that cover most of its previously reported crystal-growth parameters. Crystallization conditions, data collection, refinement and model statistics, and PDB codes are given in Table 1 ▶. All crystals were flash-cooled directly in liquid N_2_. The X-ray data were collected at 100 K and processed using *HKL*-2000 (Otwinowski & Minor, 1997 ▶) and *xia*2 (Winter, 2010 ▶), and model building and refinement were performed using the *CCP*4 suite of programs (Winn *et al.*, 2011 ▶) and *Coot* (Emsley & Cowtan, 2004 ▶). Crystal structures were solved by *MOLREP* (Vagin & Teplyakov, 2010 ▶) with a B1–B6 and B23–B30 truncated hexamer-derived insulin monomer as a model (PDB entry 1mso; Smith *et al.*, 2003 ▶) and were refined by *REFMAC* 5.8 (Murshudov *et al.*, 2011 ▶). Figures were produced using *CCP*4*mg* (McNicholas *et al.*, 2011 ▶). For structural comparisons, all insulin structures (including IR-complex-bound hormone) were superimposed on the B9–B19 C^α^ atoms using the LSQ fit option in *Coot* (Emsley & Cowtan, 2004 ▶).

## 3. Results   

### 3.1. Synthesis of the analogues and their characterization   

**Figure 2:**
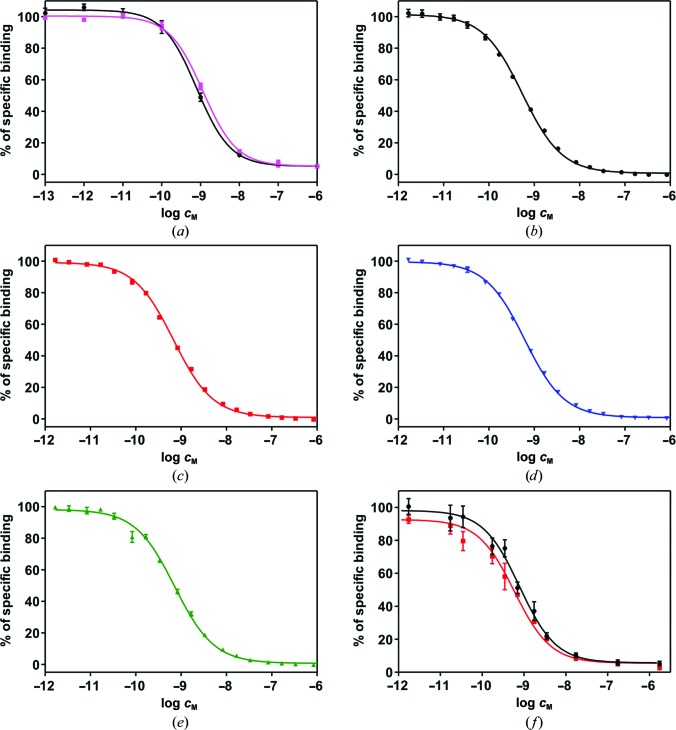
Inhibition of the binding of human ^125^I-TyrA14 insulin to IR by human insulin and insulin analogues. (*a*) Inhibition of the binding of human ^125^I-TyrA14 insulin to rat adipose plasma membranes containing both IR-A and IR-B isoforms of rat IR by human insulin (circles) and [GlyB26]-insulin (squares). (*b*–*e*) Inhibition of the binding of human ^125^I-TyrA14 insulin to the IR-A isoform of human IR in membranes of human IM-9 lymphocytes by (*b*) human insulin, (*c*) [AsnB26]-insulin, (*d*) [AspB26]-insulin and (*e*) [GlnB26]-insulin. (*f*) Inhibition of the binding of human ^125^I-TyrA14 insulin to the IR-B isoform of human IR in membranes of mouse embryonic fibroblasts by human insulin (circles) and [AsnB26]-insulin (squares).

The full-length insulin B26-site mutants AsnB26, AspB26, PheB26, GlnB26 and GlyB26 have been prepared and characterized. The synthesis and characterization of the AsnB26, AspB26 and GlnB26 analogues was inspired by the high binding affinity (193%) of [GluB26,LysB28,ProB29]-insulin (Žáková *et al.*, 2013 ▶). In the cases of [PheB26]-insulin and [GlyB26]-insulin, we aimed to investigate the structural consequences of the loss of the TyrB26 side-chain hydroxyl group or the whole TyrB26 side chain, respectively. The affinities of the analogues for the IR-A isoform (IM-9 lymphocytes) and of selected analogues for either IR-B isoform (mouse fibroblasts) or an ‘overall’ IR affinity in rat adipocyte membranes (containing both IR isoforms) were determined (Table 2 ▶ and Fig. 2 ▶). All analogues showed a relatively small drop in potency, which was maintained in the ∼70–89% range. Only the previously reported PheB26 mutant had less than 50% potency. Interestingly, the [AsnB26]-insulin increased its potency towards IR-B to 142% with a minimum loss of IR-A affinity (83%). This makes AsnB26 a unique single-mutation full-length analogue that is ∼1.7 times more IR-B selective with an overall wild-type-like IR affinity. Loss of chirality at the B26 site (GlyB26 mutant) and replacement of the phenolic Tyr side chain by a polar Gln and Asp (GlnB26 and AspB26 mutants) did not have a detrimental, or a major, effect on the affinities of these two types of analogues (∼70, ∼77 and 89%, respectively).

The new and interesting binding properties of the AsnB26 analogue prompted further investigations of its dimerization properties by ITC (Table 3 ▶). They showed a highly monomeric character of this mutant, with a *K*_diss_ (dimer↔monomer) of ∼860 µ*M*, in comparison with ∼9 µ*M* for wild-type human insulin. This indicated a rather substantial structural effect of the AsnB26 mutation on the dimer-formation capabilities of this analogue. It is more significant than just liberation of the B26 side chain (*i.e.* TyrB26) from its hydrogen-bond donor/acceptor (and aromatic/van der Waals interaction) role. Hence, the significantly smaller monomeric profile of the PheB26 mutant (*K*_diss_ of ∼27 µ*M*) than the AsnB26 analogue is more likely to be linked to release of the PheB26 side chain from the hydrogen-bond network upon losing the phenolic character of the TyrB26 site.

### 3.2. Molecular modelling   

Previous molecular-dynamics (MD) simulations of wild-type insulin and other analogues (Žáková *et al.*, 2013 ▶) underlined the importance of the water (Wat1)-mediated TyrB26–(NH)CO GlyB8 hydrogen bond for attachment of the B20–B30 chain to the insulin core and maintenance of the optimum dimer interface. Indeed, quantum-chemically computed interaction energies of the native TyrB26 side chain with the rest of insulin (including Wat1) show a strongest stabilization effect of 11–12 kcal mol^−1^ (Table 4 ▶). Nearly half of this interaction energy comes from the water-mediated TyrB26–(NH)CO GlyB8 hydrogen bond, as is revealed by calculations after the removal of Wat1 from the model. The loss of the phenolic side chain in [PheB26]-insulin also results in a ∼20% decrease (2–3 kcal mol^−1^) in the B26 side chain–insulin interaction energy. In contrast, the substitution of TyrB26 by a shorter polar side chain in the AsnB26 mutant results not only in loss of the water-mediated ‘hydrogen bridge’ but also in a repulsive character of this interaction owing to the incorporation of a polar side chain into a hydrophobic environment. Calculations starting with the structures of the *A*/*B* and *C*/*D* insulin monomers derived from the insulin dimer yielded comparable results and energy values in parallel interaction energy calculations (Table 4 ▶).

### 3.3. Crystal structures   

All of the analogues synthesized here underwent intensive crystallization screening, and the AsnB26, GlyB26 and PheB26 analogues gave high-quality crystals.

#### 3.3.1. [AsnB26]-insulin and [GlyB26]-insulin structures   

Both of these analogues crystallized as monomers, generally similar to the previously reported B26 chemically modified insulins (Jiracek *et al.*, 2010 ▶). The N-termini are present in the so-called I (intermediate) conformation, also typical for other B26-modified analogues. The most important feature of these analogues is the occurrence of the B25–B30 bend that detaches this part of the B-chain from the insulin core. In consequence, insulin site 1-related A-chain side chains are fully revealed. However, this bend differs from the ‘classical’ B26 turn (Jiracek *et al.*, 2010 ▶). Firstly, it does not maintain the typical B26-turn B24 CO–NH B26 hydrogen bond that stabilizes this turn. Instead, the B25–B30 chains in these analogues are more ‘open’, being released from this tight interaction (Figs. 1 ▶ *b* and 1 ▶ *c*); for example, the C^α^–C^α^ distance between the ProB28 residues in [NMeAlaB26]-insulin (‘classical’ B26 turn) and the AsnB26 mutant is greater than 13.5 Å. The other main difference between the ‘classical B26 turn’ and the B25–B30 chain bends in [AsnB26]- and [GlyB26]-insulins is the almost opposite direction of the B25–B30 segment to its fold in wild-type insulin and other B26-turn analogues (Figs. 1 ▶ *b* and 1 ▶ *c*). It seems that the B24–B25 peptide bond in [AsnB26]- and [GlyB26]-insulins serves as a ‘rotation axis’ for the B25–B30 chain, sweeping it almost 180° away from its wild-type direction. The ‘classical’ B26 turn only adopts directions at an angle of ∼60–70° from the wild-type insulin B20–B30 conformer (Fig. 1 ▶ *c*). Remarkably, the PheB24 residue remains relatively invariant in all of these transformations, with only PheB25 following the 180° B25–B30 chain rotation (Fig. 1 ▶ *c*). However, an impact of the crystal packing on the fold of the B24–B27 part of the analogues cannot be excluded.

The extended conformational freedom of the B25–B30 chain in the [AsnB26]- and [GlyB26]-insulins is reflected further in the different paths of their B27–B30 termini; the distance between the C^α^ atoms of the B26 residues in these analogues is greater than ∼7.4 Å. The [AsnB26]- and [GlyB26]-insulin structures show high similarity up to B26 C^α^ (only ∼0.3 Å between these atoms), but the B27–B30 chains proceed in opposite directions in these analogues. This is likely to result from the lack of conformational restraints of B26 glycine, as the crystal packings in both analogues are virtually identical.

#### 3.3.2. [PheB26]-insulin structure   

This analogue crystallized as a typical insulin dimer, with the N-termini in the I-state. Although the dimer interface is maintained in this structure, the release of the PheB26 side chain from the water-mediated (Wat1) hydrogen bond to (NH)CO of GlyB8 (typical of wild-type TyrB26) leads to relaxation of this surface. The C^α^–C^α^ distances between wild-type (TyrB26) insulin and [PheB26]-insulin increase from ∼1 Å at the B25 site to 6.3 Å at LysB29. These structural features are likely to be related to the elevated *K*_diss_ of this analogue (∼27 µ*M*; Table 3 ▶) and the ∼30% decrease in the B26 site–insulin interaction energy estimated by MD simulations (Table 4 ▶).

## 4. Discussion   

### 4.1. Structure–function correlation of the [AsnB26]-insulin analogue: potential of the B26 site for rational steering towards IR-isoform specificity   

The [GlyB26]- and [AsnB26]-insulins maintained almost native-like (∼70–83%) affinities for either the A and B isoforms in adipocytes or for the A isoform in IM-9 cells, respectively (Table 2 ▶). These are correlated with their bended B-chain C-termini, which in the case of [AsnB26]-insulin also results in a predominantly monomeric character of this analogue. The dimerization properties of [GlyB26]-insulin were not determined in this work, but it can supposed that they will be significantly lower than that of native insulin. Interestingly, [AsnB26]-insulin is more potent (140%) than native insulin for isoform B of IR (Table 2 ▶).

The IR-B:IR-A specificity ratio of insulin analogues is of considerable interest owing to IR-B being the dominant (∼90–95%) receptor isoform in the liver (Moller *et al.*, 1989 ▶). This, together with the anatomic proximity of the pancreas and the liver (short hepatic portal vein connectivity), makes the liver the major glucose homeostatic organ. The subcutaneous delivery of insulin in diabetes firstly exposes this hormone to more evenly distributed IR-A:IR-B in peripheral (for example muscle and adipose) tissue, delaying the liver response and hence increasing the risks of misbalanced glycaemic states. Liver-specific analogues may then improve the quality of glucose control and are of primordial interest (Herring *et al.*, 2014 ▶). Therefore, the enhancement of the IR-B specificity of [AsnB26]-insulin requires attention in this context, especially as it is correlated with a novel insulin conformation that is achieved by only a regular l-amino-acid substitution, without any chemical modification or the use of a d-amino acid. The currently most IR-B-specific, single-mutated, [AsnB25]-insulin analogue has already been reported by Glendorf *et al.* (2011 ▶). This mutation resulted in a doubling of the IR-B specificity of this analogue. It was doubled further by additional HisA8 and Asp/GluB27 mutations. However, it was the B25 site that was identified as the key determinant of the IR-B specificity of insulin. Therefore, the enhanced IR-B selectivity of the AsnB26 mutant is quite unexpected as it was achieved by mutation of the ‘IR-irrelevant’ B26 site. As the B26 side chain is not needed for direct insulin–IR binding, the gain in IR-B specificity is likely to originate from the conformational (‘entropic’) effect of this mutation. Therefore, the B26 site opens new possibilities for the exploration of IR-B-specificity oriented mutations, expanding a similar potential to the B25 site.

**Figure 3:**
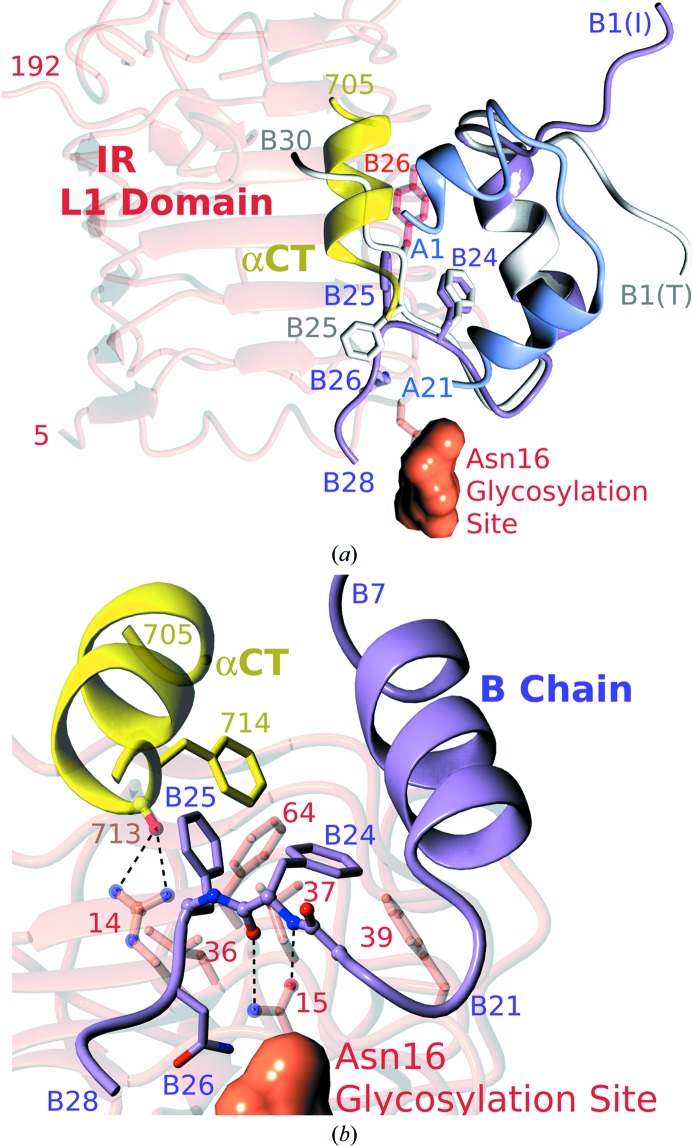
(*a*) Fitting of the [AsnB26]-insulin B26-turn-like structure (lilac) to the insulin–IR complex. The modelled native insulin B-chain is shown in white and the modified TyrB26 site is shown in red. The colour code is as in the other figures. (*b*) The B26-turn-like conformation poises the [AsnB26]-insulin B-chain to fulfil hydrophobic and hydrogen-bond (dashed lines) interactions with some key IR residues (O atoms in red, N atoms in blue). The hydrogen-bond capping of the Val713 CO group by the important Arg14 (that must be bypassed by the B27–B30 chain) is also indicated.

It has to be stressed that [AsnB26]-insulin was screened for crystallization against a very broad range of conditions that also included parameters very similar to those reported for the crystal structures of other IR-B-specific mutants (HisA8/AsnB25, HisA8/AsnB25/GluB27 and HisA8/HisB25/GluB27; Glendorf *et al.*, 2011 ▶). Moreover, the AsnB25 mutation (similar to all other substitutions performed in that work) did not cause any structural perturbation in these analogues, which were capable of forming typical insulin dimers. Therefore, the structure of [AsnB26]-insulin with the characteristic B26 bend is likely not to be accidental but represents a favourable and stable conformation of this analogue. Hence, the likelihood of an ‘entropic/structural’ effect of the Asn at the B26 site is reinforced. The putative intramolecular side-chain-mediated hydrogen bonds between the B25 and B27 sites postulated by Glendorf *et al.* (2011 ▶) cannot be fulfilled in the AsnB26 mutant as its PheB25 side chain is close only to the PheB24 [van der Waals (∼3.5 Å) contact] (Fig. 3*b* ▶). Hence, the wild-type ‘B24down-B25up-B26down’ directionality of the side chains seen in the native insulin storage-form B24–B30 β-strand is practically reversed here. Moreover, the peptide NH of AsnB26 increases the stability of the B26 bend through a hydrogen bond to OD1 of the A-chain C-terminal AsnA21. The side chain of AsnB26 is also near enough (∼3.5 Å) to the CO group of ThrB27 to make another hydrogen bond that would stabilize this region even further. Therefore, the introduction of the Asn residue in the B26 site enhances the stability of the newly formed, bent insulin conformer. As such a contribution was not observed in AsnB25-containing analogues, the origins of the IR-B specificity in these analogues may differ, being more entropic/conformational in the AsnB26 mutant and more enthalpic/side-chain interaction-specific in the AsnB25-derived insulins.

### 4.2. IR-related physiological relevance of the conformation of the AsnB26 insulin analogue   

The [AsnB26]- and [GlyB26]-insulin structures indicate that wild-type insulin can attain the B26-turn-like structure. They also expand the conformational landscape of the B25–B30 chain (Figs. 1 ▶ *b* and 1 ▶ *c*). The previously described B26 turn (Jiracek *et al.*, 2010 ▶) can therefore be considered to be a structural template that is modulated, and easily induced, by various substitutions and modifications. This structural behaviour of the B25–B30 segment of the B-chain reflects its inherent plasticity and is documented further by ITC dimerization (Table 3 ▶) and MD studies (Table 4 ▶). Comparison of the [GlyB26]- and [AsnB26]-insulin structures with the previously reported high-affinity B26-turn-containing truncated insulins (Jiracek *et al.*, 2010 ▶) suggests that the direction of the B-chain in the B26-shortened analogues may vary without having a large detrimental effect on their IR affinity. However, the full-length AsnB26 and GlyB26 analogues prefer the extreme end of this conformational spectrum (Figs. 1 ▶ *b* and 1 ▶ *c*), likely reflecting the need for structural accommodation of the remaining C-terminal B27–B30 segment.

All of these structural and functional data narrow the conformational flexibility of the insulin B-chain to its B25–B26 sites, indicating the readiness of native insulin to adopt B26-turn-like structures.

Remarkably, the physiological significance of the B26-turn-like/bend conformation in [AsnB26]-insulin is augmented further by its direct comparison with the insulin–IR complex. An unmodelled and non-optimized superposition of this analogue onto receptor-bound insulin places the B21–B30 chain of this analogue in a close-to-optimum complex-like structural environment (Fig. 3 ▶ *a*). Only the B25 side chain and the B27–B30 chain would require small (easily achievable, for example by a slightly different rotamer of PheB25) readjustments in this model to avoid clashes with the receptor (mainly with the surroundings of Arg14). This, even ‘approximate’, insulin–IR fitting supports the physiological relevance of the B26-turn-like bend as some of the important side chains of insulin (PheB24 and PheB25) are primed to be engaged in this model in hydrophobic interactions with the key IR residues (Phe39, Phe64 and Phe714; Fig. 3 ▶ *b*). The constellation of these side chains also agrees with insulin–IR photo-cross-linking studies as B24 and B26 face the L1 domain, while PheB25 can make contacts with both the αCT segment and the L1 domain (Xu *et al.*, 2004 ▶, 2009 ▶). Moreover, the B26 bend in AsnB26-insulin positions the PheB24 peptide atoms to fulfil potential hydrogen bonds with the important IR Asn15, also enabling the bypassing of the IR Asn16 glycosylation site (Fig. 3 ▶ *b*).

## 5. Conclusions   

In summary, the [AsnB26]- and [GlyB26]-insulin analogues are the first naturally substituted insulins with a stable conformation that differs from that in all known structures of the native hormone. This indicates that human insulin may attain a B26-turn-like conformation upon IR binding, which is documented here by a remarkably overall good fit of the presented structures into the currently described (B21–B30-deficient) insulin–IR complex. This conformation (especially at the B27–B30 terminus) must be tuned further by IR components that have not yet been characterized in the current insulin–IR complex structures: the full αCT segment (especially with exon 11 present in the longer IR-BαCT) and IR site 2 (for example the FnIII-1 and FnIII-2 IR domains). However, the main features of [AsnB26]-insulin (and to some extent [GlyB26]-insulin as well), such as the preservation of the PheB24 conformation and the B26-turn-like bend, may reflect the actual direction of the insulin B-chain on the IR.

Moreover, the unexpected but significant IR-B selectivity of the AsnB26 mutant that is correlated with the characteristic bend of the B-chain of the hormone may give the first structural insight into the structural origins of differential insulin signalling through the IR-B and IR-A isoforms. Although it is far too early to derive any details of this phenomenon from the structure and properties of [AsnB26]-insulin, they are intriguing and may stimulate a more rational approach towards clinically relevant, IR-B/liver-specific novel insulins.

## Supplementary Material

PDB reference: [AsnB26]-insulin, 4ung

PDB reference: [GlyB26]-insulin, 4unh

PDB reference: [PheB26]-insulin, 4une

## Figures and Tables

**Table 1:** Data-collection and refinement statistics Values in parentheses are for the highest resolution shell. All X-ray data were collected from only one crystal.

	[AsnB26]-insulin	[GlyB26]-insulin	[PheB26]-insulin
PDB code	4ung	4unh	4une
Beamline	I02, DLS†	ID29, ESRF‡	ID23, ESRF‡
Detector	ADSC	ADSC	MAR225
Wavelength (Å)	0.9795	0.9763	0.8726
Space group	*P*4_1_2_1_2	*I*4_1_22	*P*2_1_2_1_2_1_
Unit-cell parameters			
*a* (Å)	45.63	45.52	44.30
*b* (Å)	45.63	45.52	46.19
*c* (Å)	117.72	117.41	51.76
α = β = γ (°)	90.0	90.0	90.0
Resolution (Å)	45.63–1.81 (1.85–1.81)	42.45–2.75 (2.80–2.75)	50.0–1.59 (1.62–1.59)
*R*_merge_	0.110 (0.817)	0.138 (0.345)	0.081 (0.537)
〈*I*/σ(*I*)〉	19.1 (4.2)	12.0 (3.0)	20.3 (5.7)
Completeness (%)	99.6 (99.6)	94.0 (59.3)	100.0 (100.0)
Multiplicity	14.2 (15.6)	12.7 (9.1)	7.1 (7.1)
Wilson *B* (Å^2^)	19.30	62.9	14.82
Refinement			
Resolution (Å)	36.06–1.81	42.45–2.75	34.15–1.59
No. of reflections	11457	1617	13606
*R*_work_/*R*_free_	0.174/0.220	0.237/0.355	0.153/0.180
No. of atoms			
Total	947	364	963
Protein	813	354	792
Ligand/ion	5	5	10
Water	129	5	161
*B* factors (Å^2^)			
Protein	19.95	45.64	11.8
Ligand/ion	13.27	34.43	30.4
Water	30.37	25.55	21.9
R.m.s. deviations			
Bond lengths (Å)	0.019	0.012	0.023
Bond angles (°)	1.782	1.518	2.118
Ramachandran plot (%)			
Preferred/allowed	98.6/1.4	91.1/8.9	98.9/1.1
Crystallization§	0.035 *M* (NH_4_)_2_SO_4_ pH 4.0 [(NH_4_)_2_SO_4_ stock solution adjusted to pH 4.0 with H_2_SO_4_]	0.08 *M* Na_2_SO_4_ pH 4.0 (Na_2_SO_4_ stock solution adjusted to pH 4.0 with H_2_SO_4_)	0.15 *M* Na_2_SO_4_ pH 4.0

† Diamond Light Source, Didcot, England.

‡ European Synchrotron Radiation Facility, Grenoble, France.

§ 10 mg ml^−1^ insulin in 0.025 *M* HCl, hanging-drop method, 1:1 or 1:2 protein:well drop ratio, 1–2 µl drops, no cryoprotection, direct flash-cooling in liquid N_2_.

**Table 2:** Values of *K*_d_, IC_50_ and the relative binding affinities of human insulin and the insulin analogues reported in this work

Protein	*K*_d_ ± SE for human IR-A in IM-9 lymphocytes (n*M*) (*n*)	Potency† (%)	*K*_d_ ± SE for human IR-B in mouse fibroblasts (n*M*) (*n*)	Potency (%)	IC_50_ ± SE‡ for both rat IR-A and IR-B in rat adipocytes (n*M*) (*n*)	Potency (%)
Human insulin	0.39 ± 0.01 (6)	100	0.68 ± 0.08 (4)	100	0.81 ± 0.08 (6)	100
[AsnB26]-insulin	0.47 ± 0.01 (4)	83	0.48 ± 0.07 (3)	142	nd§	—
[AspB26]-insulin	0.44 ± 0.01 (4)	89	nd	—	nd	—
[GlnB26]-insulin	0.51 ± 0.02 (4)	77	nd	—	nd	—
[GlyB26]-insulin	nd	—	nd	—	1.16 ± 0.12 (5)	70
[PheB26]-insulin	nd	—	nd	—	nd	46¶

† Relative receptor-binding affinity (potency) is defined as (IC_50_ or *K*_d_ of human insulin/IC_50_ or *K*_d_ of analogue) × 100.

‡ IC_50_ values represent the concentrations of insulin or the analogues that cause half-maximal inhibition of binding of human ^125^I-TyrA14 insulin to IR. Each value represents the mean ± SE of multiple determinations (*n*).

§ Not determined in this study.

¶ From Jiracek *et al.* (2010 ▶) for both rat IR-A and IR-B in rat adipocytes.

**Table 3:** ITC analyses of the dimerization capabilities of insulin and insulin analogues

Insulin	*K*_diss_ (µ*M*)	Δ*H*°_diss_ (kJ mol^−1^)	Δ*G*°_diss_ (kJ mol^−1^)	Δ*S*°_diss_ (J K^−1^ mol^−1^)
Human insulin (*n* = 8)†	8.81 ± 1.05	56.93 ± 2.56	28.95 ± 0.28	93.90 ± 8.15
[AsnB26]-insulin (*n* = 3)	865 ± 309	8.95 ± 1.53	17.76 ± 0.82	−29.58 ± 7.87
[PheB26]-insulin (*n* = 3)	27.2 ± 7.4	37.32 ± 5.60	26.21 ± 0.63	32.28 ± 17.06

† The experimental values for human insulin are from Antolikova *et al.* (2011 ▶).

**Table 4:** Interaction energies (kcal mol^−1^) between the side chains of B26 and the rest of insulin (values are relative to GlyB26, which has a value of 0)

Human insulin B26 residue	*A*/*B* monomer	*C*/*D* monomer
Asn	1.3	2.5
Phe	−9.0	−9.3
Tyr	−7.3	−6.5
Tyr†	−11.4	−12.0

† Wat1 included as part of insulin.
